# Asymmetrically Wettable, PET/PA6, Hollow, Segmented-Pie, Microfiber Nonwovens for a Synthetic Leather Base

**DOI:** 10.3390/molecules29122891

**Published:** 2024-06-18

**Authors:** Baobao Zhao, Chunbiao Liu, Zhen Wang, Quan Feng, Xu Han, Jin Zhang, Chenggong Hu, Dongxu Han

**Affiliations:** 1 School of Textile and Garment, Anhui Polytechnic University, Wuhu 241000, China; 15080203207@163.com (C.L.); wangzhen@ahpu.edu.cn (Z.W.); fengquan@ahpu.edu.cn (Q.F.); hanxu@ahpu.edu.cn (X.H.); zhangjin@ahpu.edu.cn (J.Z.); 2 Advanced Fiber Materials Engineering Research Center of Anhui Province, Anhui Polytechnic University, Wuhu 241000, China; 3 School of Chemical and Environmental Engineering, Anhui Polytechnic University, Wuhu 241000, China; 4 College of Textile and Clothing Engineering, Soochow University, Suzhou 510632, China; handongxuahpu@163.com

**Keywords:** microfiber nonwovens, synthetic leather, metal–organic framework materials, asymmetric wettability, absorption–desorption isotherms

## Abstract

PET/PA6, hollow, segmented-pie, microfiber nonwovens (PET/PA6 HSMNs) play an important role in a microfiber, synthetic leather base. Most of the current PET/PA6 HSMNs generally lack in hygiene performance. Moreover, there is an urgent need for the asymmetric wettability of PET/PA6 HSMNs to ensure the comfort of clothing. In this work, a novel, asymmetrically wettable, PET/PA6 HSMN with a gradient structure is proposed, which can regulate liquid adsorption and directional transport. An MOF-303 was successfully synthesized and loaded onto the PET/PA6 HSMN, which had been pre-treated with gradient hydrolysis under sulfuric acid. The droplet quickly permeated the modified PET/PA6 HSMN, and the droplet disappearance time decreased to 62.40 ms. The liquid strikethrough time of the modified PET/PA6 HSMN reached 5.16 s. The maximum adsorption capacity of the modified PET/PA6 HSMN was 68.161 mg/g, which was improved by 122.83%. In addition, the air permeability of the pre-treated PET/PA6 HSMN increased from 308.70 mm/s to 469.97 mm/s, with the sulfuric acid concentrations increasing from 0% to 20%, and the air permeability of the modified PET/PA6 HSMN decreased gradually from 247.37 mm/s to 161.50 mm/s. Furthermore, the tensile strength of the modified PET/PA6 HSMN treated with sulfuric acid and MOF-303 was also obviously enhanced compared with the PET/PA6 HSMN treated with pure sulfuric acid. This PET/PA6 HSMN, with asymmetric wettability, owing to its high hygiene performance and water transport capabilities, is promising and able to extend the application of a microfiber synthetic leather base for clothing.

## 1. Introduction

The development and research of high-performance, synthetic leather have become one of the key focuses in the global leather industry due to the limited resources of natural leather. Microfiber synthetic leather is a composite material composed of microfiber nonwoven materials (as a supportive layer) and elastic resin (as a coating or filling material). From its inception to development, it has always imitated natural leather. As a product of structural biomimicry, microfiber synthetic leather achieves the transition from “imitation” to “simulation” of natural leather by applying and modifying microfibers (mimicking the diameter of collagen fibers in natural leather), nonwoven materials (mimicking the three-dimensional network structure of natural leather), and polyurethane resin (mimicking the release structure). It is currently the new generation of high-end synthetic leather in the world, with its structure and performance being closest to natural leather, and has been applied in clothing, accessories, footwear, bags, sofas, car interiors, and other fields. However, compared to natural leather, microfiber synthetic leather generally lacks in hygiene performance, especially in terms of moisture absorption and breathability. This leads to the inability of human sweat to be expelled in a timely manner, affecting the comfort of clothing and significantly restricting its extensive application in the clothing industry, thereby constraining the development of the synthetic leather industry. Therefore, developing microfiber synthetic leather with high moisture absorption and breathability has become a necessary consideration in the development of the leather industry, attracting widespread attention from countries around the world.

Existing microfiber manufacturing methods include direct spinning [1,2], conjugate spinning (islands-in-sea type [3,4,5], split type [6,7,8], and multi-layer type [9,10]), and random spinning (melt blowing [11,12,13], spun bonding [14,15,16], flash spinning [17,18], and electrospinning [19,20,21]). Among them, the islands-in-sea bicomponent spinning is a technique that can rapidly and efficiently prepare microfiber nonwovens, which has received widespread attention in the industry and has become the main approach for producing microfiber synthetic leather bases. However, the moisture absorption and permeability of microfiber synthetic leather bases prepared by this method are poor, and the reduction process involves serious pollution from alkaline solution and toluene. Compared with the islands-in-sea type, the split spinning type utilizes physical extrusion shear to break the interface between two different components, thereby producing the ultimate microfibers. Split-type microfiber nonwovens have numerous capillaries due to the splitting of conjugate filaments. These nonwovens are composed of synthetic filaments that usually have hydrophobic characteristics, so they rapidly absorb and transport moisture because of these capillaries [22]. Our research group analyzed the microstructure and properties of PET/PA6, hollow, segmented-pie, microfiber nonwovens (PET/PA6 HSMNs) in detail and verified their feasibility of use in a microfiber synthetic leather base. The results showed that the average diameter of the bicomponent composite fibers prepared by this technology was 15 μm, and the average diameter of the microfibers formed after hydroentanglement was 5 μm (similar to the diameter of collagen fibers), but the moisture absorption and permeability of the nonwovens were far from meeting the standards of natural leather. We used the electrospinning method to prepare a series of polyacrylonitrile (PAN) nanofibers with diameters of 200 nm, 450 nm, and 900 nm. By blending, carding, and hydroentangling with hollow, segmented-pie, bicomponent, composite fibers, micro- and nanomicrofiber synthetic leather bases with improved water absorption performance and static water vapor transmission rate were obtained, increased by 768.99% and 28.20%, respectively. We successfully prepared thermoplastic polyurethane (TPU)/sulfonated polysulfone (SPSf)-electrospun nanofibers (average diameter of 120 nm) and applied them to a microfiber synthetic leather base. With an increase of TPU/SPSf nanofiber content from 0% to 30 wt%, the contact angles of the synthetic leather base decreased gradually from 111.64 degrees to 67.07 degrees, leading to 55.19% improvement in the water vapor transmission rate (from 2868.96 g/(m^2^·24 h) to 4452.24 g/(m^2^·24 h)) and 26.25% improvement in moisture absorption (from 628.70% to 793.75%). In addition, we used one-step and three-step methods to prepare a series of gradient-structured PET/PA6 HSMNs. The construction of a gradient structure increased the moisture permeability of the nonwoven leather substrate from 3296.11 g/(m^2^·24 h) to 4243.82 g/(m^2^·24 h). Therefore, it could be seen that the existing fiber modification and structural design could not fundamentally solve the problem of poor moisture absorption and permeability of PET/PA6 HSMNs. Therefore, it is an inevitable trend to explore a new material or new method to enhance the moisture absorption and permeability performance of PET/PA6 HSMNs through multiple mechanisms.

Metal–organic framework materials (MOFs) are a novel type of material composed of periodic connections between metal ions or metal clusters and organic ligands, exhibiting both high crystallinity and porosity. By altering metal and organic ligands, the shape, size, and functionality of a micropore system can be precisely controlled, influencing its adsorption capabilities for certain molecules. MOFs have demonstrated significant potential in areas such as gas separation, gas storage, catalysis, and sensing. In recent years, researchers have addressed the challenge of the stability of MOFs in water and discovered that designing the structure of MOFs can result in excellent water adsorption performance. Consequently, water vapor capture has become an attractive application direction for MOFs. Amandine et al. [23] developed a selective MOF material (KAUST-8), capable of selectively removing water vapor from gas streams containing CO_2_, N_2_, CH_4_, and typical higher hydrocarbons, as well as selectively removing H_2_O and CO_2_ from N_2_ streams. Chen et al. [24] synthesized a series of MOFs based on trivalent metals (iron, chromium, and scandium) and rigid triangular prism ligands, such as NU-1500. This MOF has a high porosity, excellent water stability, and outstanding water vapor absorption and release capabilities. Kim et al. [25] and Fathieh et al. [26] developed two materials (MOF-801 and MOF-303) capable of spontaneously capturing water vapor from the air. These materials were assembled into water collectors, effectively absorbing water from atmospheric moisture with a high water stability and absorbent performance. Moreover, these water-absorbing MOF materials exhibit reversibility: they absorb water when humidity is high and release water when humidity is low. It is evident that the innovative applications of MOF materials in water vapor capture open up a new pathway and perspective for enhancing the moisture absorption performance of a microfiber synthetic leather base. 

Therefore, this project aims to address the issue of insignificant improvement in the moisture absorption and breathability of a microfiber synthetic leather base through the existing research. Firstly, a multi-stage spray method using sulfuric acid (H_2_SO_4_) was employed to modify PET/PA6 HSMNs through a gradient. Then, an in situ growth method was utilized to surface-immobilize highly water-absorbent MOF-303, achieving asymmetric distribution of material hydrophilic properties. The mechanism of moisture absorption and breathability of an asymmetric microfiber synthetic leather base was studied. This research is expected to solve the long-standing problem of the poor moisture absorption and breathability performance of microfiber synthetic leather and lay a foundation for its wide application in the field of clothing.

## 2. Results and Discussion

### 2.1. Micromorphology Analysis

**Figure 1:**
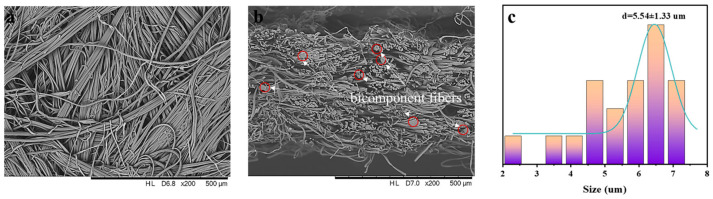
(**a**) Surface SEM image of the untreated PET/PA6 HSMN. (**b**) Cross-section SEM image of the untreated PET/PA6 HSMN. (**c**) Mean microfiber diameter of the untreated PET/PA6 HSMN.

Figure 1a,b show the SEM images of the surface and cross-section of an untreated PET/PA6 HSMN. It can be seen that the untreated PET/PA6 HSMN had a three-dimensional network structure. Most of the PET/PA6 bicomponent fibers were split into microfibers, and the degree of splitting was about 77.00%. The mean fiber diameter after splitting was 5.54 μm (see Figure 1c), which was similar to monofilaments of natural leather.

**Figure 2:**
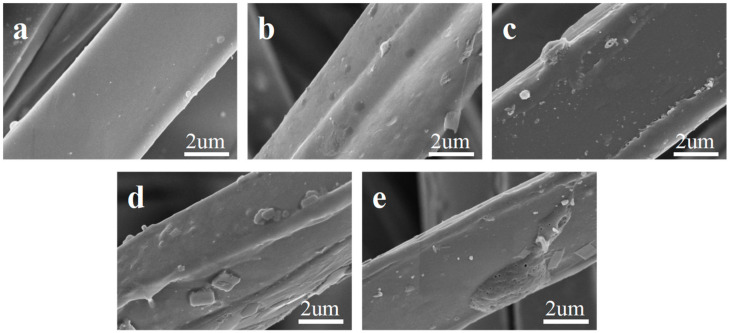
SEM images of microfiber nonwovens treated with sulfuric acid at different concentrations: (**a**) untreated; (**b**) 5%; (**c**) 10%; (**d**) 15%; (**e**) 20%.

SEM images of nonwovens modified with sulfuric acid at different concentrations are shown in Figure 2. According to Figure 2a, the microfiber surface of the untreated nonwoven was very smooth. When the concentration of sulfuric acid modification was 5%, the surface of the microfiber had micropits and was longitudinally uneven, as shown in Figure 2b. With sulfuric acid treatment concentration increasing to 10–15%, the roughness of the fiber surface further increased, as shown in Figure 2c,d. When the concentration of sulfuric acid modification was 20%, the shape of the fiber surface changed and micropores gradually appeared, as depicted in Figure 2e. In addition, after sulfuric acid was sprayed onto the surface of the nonwoven fabric, it transferred to the interior of the nonwoven fabric under the action of gravity to modify the fibers inside and achieve an asymmetric structure. The nonwoven fabric that had been sprayed was naturally air-dried, so the time for the sulfuric acid treatment was considered to be infinite. 

**Figure 3:**
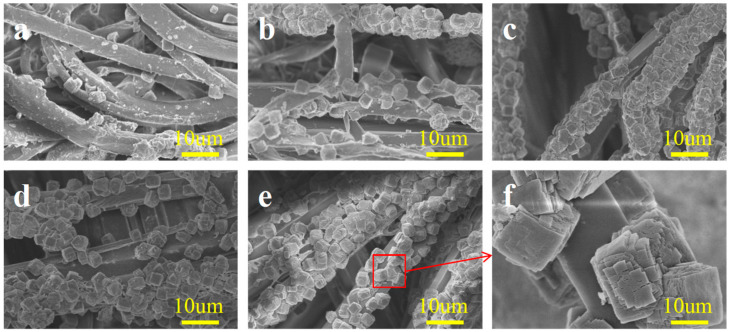
SEM images of microfiber nonwovens treated with sulfuric acid at different concentrations and MOF-303: (**a**) untreated; (**b**) 5%; (**c**) 10%; (**d**) 15%; (**e**) 20%; (**f**) MOF-303.

According to Figure 3a, it can be seen that using the in situ growth method to directly load MOF-303 on untreated PET/PA6 HSMNs was very unsatisfactory. Only a small number of MOF-303 crystals were bound to the microfibers. In contrast, as shown in Figure 3b–e, nonwovens pre-treated with sulfuric acid showed a significant increase in the number of loaded MOF-303 crystals, and the higher the sulfuric acid concentration, the more crystals were loaded. This may be because the surface of untreated PET/PA6 HSMNs is smooth, making it difficult for MOF crystals to be directly loaded on their surface. After sulfuric acid modification, the roughness of the microfibers increased, and the fluctuation on the surface of microfibers created a good space for the loading of MOF-303. On the other hand, after sulfuric acid treatment, more hydrophilic functional groups such as –COOH and –NH_2_ were exposed on the surface of PA6 microfibers due to hydrolysis. These groups will promote the growth of MOF-303. From Figure 3f, it can be clearly seen that the synthesized MOF-303 had an approximately cubic, porous, crystal structure with numerous and continuous voids, which is conducive to the subsequent filling of water molecules.

The EDS data of the microfiber nonwovens treated with sulfuric acid at different concentrations and MOF-303 are presented in Table 1. As shown in the table, the surface of the microfiber nonwovens was mainly composed of C, O, N, and Al elements. The microfiber nonwovens without sulfuric acid treatment underwent direct in situ growth of the MOF-303, resulting in low Al content (0.58 atomic percent). After preprocessing with sulfuric acid at different concentrations, the Al content increased to 4.56%, indicating that more MOF-303 was successfully synthesized on the surface of microfiber nonwovens.

### 2.2. Wetting Behavior

**Figure 4:**
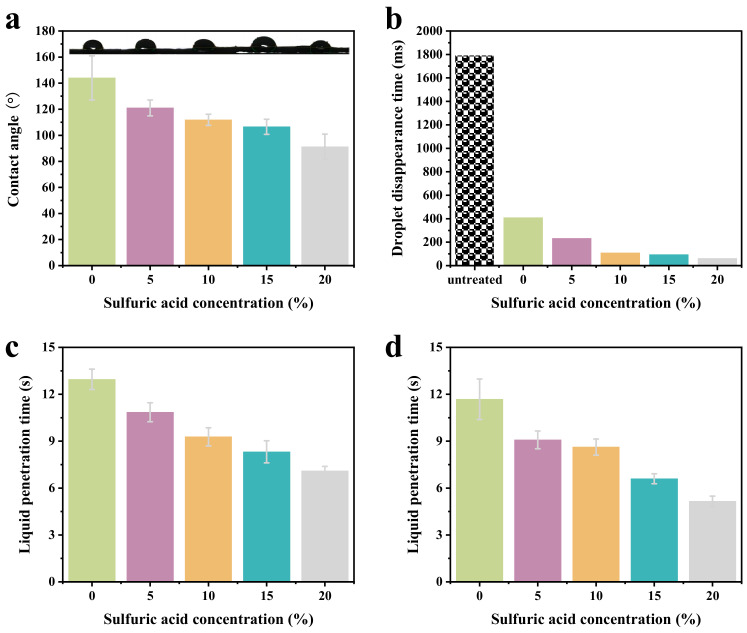
(**a**) Contact angles of microfiber nonwovens treated with sulfuric acid at different concentrations. (**b**) Droplet disappearance times of microfiber nonwovens treated with sulfuric acid and MOF-303. (**c**) Liquid strikethrough times of microfiber nonwovens treated with sulfuric acid at different concentrations. (**d**) Liquid strikethrough times of microfiber nonwovens treated with sulfuric acid and MOF-303.

The water contact angles of the prepared microfiber nonwovens treated with sulfuric acid at different concentrations are shown in Figure 4a,b. As shown in Figure 4a, the surfaces of the untreated microfiber nonwovens exhibited hydrophobicity with a WCA of 144.02°, owing to the hydrophobic PET microfibers and PA6 microfibers and the special dense network structure of the microfiber nonwovens. However, after sulfuric acid modification, the contact angle of the microfiber nonwovens significantly decreased. With an increase of sulfuric acid concentrations from 5% to 20%, their water contact angle decreased gradually from 120.98° to 91.20°. This is because PA6 is unstable to acids. Due to hydrolysis reactions, the amide bonds of PA6 molecules break, forming more hydrophilic functional groups.

The droplet disappearance times of the prepared microfiber nonwovens treated with sulfuric acid and MOF-303 are shown in Figure 4b. For hydrophobic, untreated, microfiber nonwovens, the water contact angle changed only slightly after a water droplet was dropped on the surface. The droplet disappearance time was 1792.10 ms. When the MOF-303 was coated on the microfiber nonwovens treated with sulfuric acid, a droplet quickly permeated the modified microfiber nonwovens, and the droplet disappearance time decreased to 62.40 ms. It can be seen that the modified PET/PA6 HSMNs became a superhydrophilic fabric (a superhydrophilic fabric means that its contact angle is less than 10° within 10 s). Compared with the previous literature, the current article presents a method that significantly improved hydrophilic performance of PET/PA6 HSMNs for their application in synthetic leather. 

The liquid strikethrough time of microfiber nonwovens was investigated. As shown in Figure 4c, the liquid strikethrough time of microfiber nonwovens treated with sulfuric acid at different concentrations decreased from 12.96 s to 7.10 s. The microfiber nonwovens treated with sulfuric acid and MOF-303 took less time of 11.68 s to 5.16 s, respectively, to transport liquid from the upper layer to the inner layer (see Figure 4d). The results indicate that after sulfuric acid and MOF modification, the hydrophilicity of the microfiber nonwovens increased, promoting the penetration of liquids.

### 2.3. Water Vapor Adsorption and Desorption

**Figure 5:**
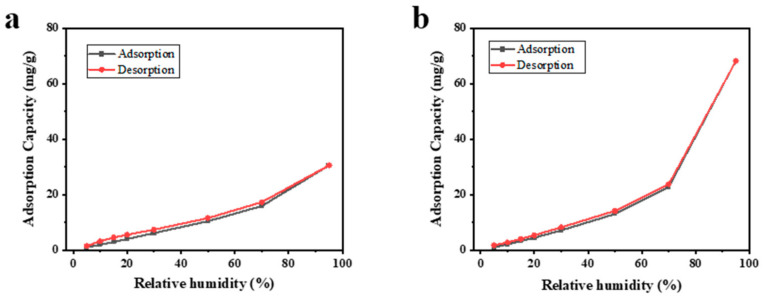
Absorption–desorption isotherms of water vapor on microfiber nonwovens: (**a**) untreated microfiber nonwovens; (**b**) microfiber nonwovens treated with sulfuric acid of 20% and MOF-303.

Water vapor adsorption and desorption experiments have been used to determine the water adsorption mechanism of common adsorbents in porous materials. The absorption–desorption isotherms of microfiber nonwovens are considered an effective method to distinguish water molecules in different adsorption sites. Figure 5 displays the absorption–desorption isotherms of untreated microfiber nonwovens and microfiber nonwovens treated with sulfuric acid of 20% and MOF-303. When the relative humidity increased, the adsorption capacity of the two samples continued to rise, and their trends of change were similar. The increase in adsorption capacity was smaller at lower relative humidity, but when the relative humidity exceeded 70%, the samples showed strong adsorption ability. By comparing the results of the two samples, it was found that the adsorption capacity of the microfiber nonwovens treated with sulfuric acid and MOF-303 was larger. At a relative humidity of 95%, the maximum adsorption capacity of untreated microfiber nonwovens was 30.589 mg/g (see Figure 5a), while the maximum adsorption capacity of modified microfiber nonwovens was 68.161 mg/g (see Figure 5b), which is an increase of 122.83%. Moreover, the modified microfiber nonwovens reached saturation level at each partial pressure point in a shorter time.

### 2.4. Air Permeability

**Figure 6:**
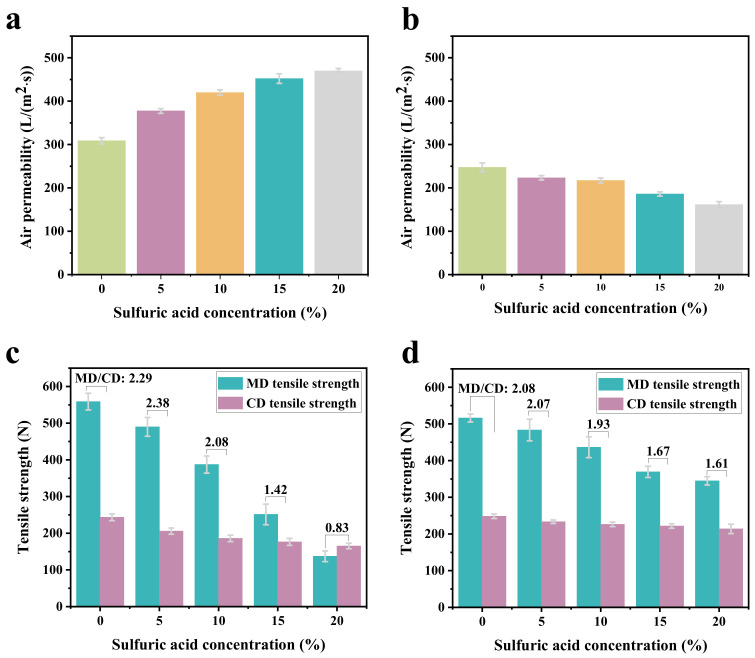
(**a**) Air permeability of microfiber nonwovens treated with sulfuric acid at different concentrations. (**b**) Air permeability of microfiber nonwovens treated with sulfuric acid and MOF-303. (**c**) Tensile strength of microfiber nonwovens treated with sulfuric acid at different concentrations. (**d**) Tensile strength of microfiber nonwovens treated with sulfuric acid and MOF-303.

It can be seen from Figure 6a that the air permeability obtained from the microfiber nonwovens treated with sulfuric acid of 5% was 377.47 mm/s, which was higher than that obtained from untreated microfiber nonwovens (308.70 mm/s). Furthermore, the air permeability of the microfiber nonwovens increased from 419.87 mm/s to 469.97 mm/s with the sulfuric acid concentrations increasing from 10% to 20%. This was mainly due to the hydrolysis of some PA6 microfibers after treatment of the nonwovens with sulfuric acid, resulting in a reduction in fiber entanglement and an increase in porosity. 

The air permeability of microfiber nonwovens treated with sulfuric acid at different concentrations and MOF-303 are shown in Figure 6b. From the figure, it can be seen that the air permeability of the microfiber nonwovens treated with MOF-303 shows a value of 247.37 mm/s. With the increase of sulfuric acid concentrations from 5% to 20%, their air permeability decreased gradually from 223.10 mm/s to 161.50 mm/s. This implies that the loading of MOF-303 had an obvious influence on the air permeability of the microfiber nonwovens. This may have been because the porosity of the microfiber nonwovens decreasing after loading with MOF-303.

### 2.5. Tensile Strength

It can be seen from Figure 6c that the MD tensile strength of the microfiber nonwovens treated with sulfuric acid at different concentrations was higher than that of the CD tensile strength. This is mainly related to the fiber distribution of microfiber nonwovens. Moreover, sulfuric acid modification has a great effect on the tensile strength of nonwovens, especially their MD tensile strength. With the increase of sulfuric acid concentrations from 0% to 20%, the tensile strength of the microfiber nonwovens in MD and CD gradually decreased from 558.77 N and 243.68 N to 137.12 N and 165.32 N, respectively. The ratios of MD tensile strength and CD tensile strength were 2.29, 2.38, 2.08, 1.42, and 0.83. This may be because, as the concentration of sulfuric acid increased, the number of fiber macromolecular breaks increased, and the effective molecular chain of the bearing force became shorter, resulting in a corresponding decline in the ability of the fiber to resist external damage. 

Figure 6d illustrates the effect of treatment with sulfuric acid and MOF-303 on the tensile strength of the microfiber nonwovens. As shown in Figure 6d, with the increase of sulfuric acid concentrations from 0% to 20%, the MD tensile strength of the microfiber nonwovens loaded with MOF-303 gradually decreased from 516.10 N to 344.75 N, and the CD tensile strength of microfiber nonwovens loaded with MOF-303 gradually decreased from 248.28 N to 213.90 N. Clearly, the tensile strength of the microfiber nonwovens loaded with MOF-303 still decreased with the increase in sulfuric acid modification concentration, but compared with the modification only with sulfuric acid, the loading of MOF-303 played a role in repairing and alleviating, and the tensile strength decreased significantly more slowly. Compared with the MD tensile strength, the CD tensile strength was lower, and the degree of damage was smaller. This is most likely due to the fact that the loaded MOF-303 nanocrystals filled the fiber surface damaged by sulfuric acid. When the nonwovens were stretched by external forces, the effective cross-sectional area of the stressed fibers increased, and the ability of the nonwovens to resist external forces was improved compared with the nonwovens modified by pure sulfuric acid.

## 3. Materials and Methods

### 3.1. Materials

PET/PA6, hollow, segmented-pie, microfiber nonwovens (PET/PA6 HSMN, 50/50 composition ratio, 120 g/m^2^; Sanjiang Enterprise Co., Ltd., Jian, China), sulfuric acid (H_2_SO_4_; Sinopharm Chemical Reagent Co., Ltd., Shanghai, China), aluminum chloride (AlCl_3_; Shanghai Macklin Biochemical Technology Co., Ltd., Shanghai, China), 3,5-pyrazoledicarboxylic acid (H_3_PDC·H_2_O; Shanghai Yuanye Bio-Technology Co., Ltd., Shanghai, China), sodium hydroxide (NaOH; Shanghai Macklin Biochemical Technology Co., Ltd., Shanghai, China), and methanol (CH_3_OH; Shanghai Macklin Biochemical Technology Co., Ltd., Shanghai, China) were used.

### 3.2. Preparation of Asymmetrically Wettable PET/PA6 HSMNs

**Figure 7:**
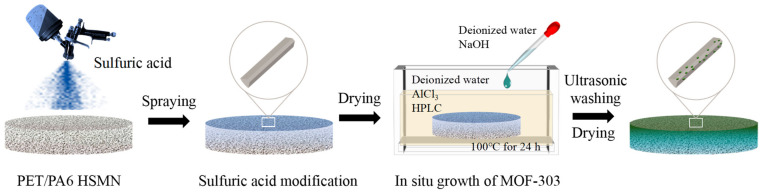
Preparation of asymmetrically wettable PET/PA6 HSMNs by the in situ growth method.

Figure 7 shows the preparation process of the asymmetrically wettable PET/PA6 HSMNs by the in situ growth method. The preparation process can be divided into two steps.

Sulfuric acid modification. Firstly, the PET/PA6 HSMNs were cut into a circle with a diameter of 300 mm. Then, sulfuric acid with a concentration of 98% was diluted to 5%, 10%, 15%, and 20%, respectively. The four concentrations of diluted sulfuric acid solutions were sprayed onto the samples. The amount of sprayed sulfuric acid was controlled at 5.0 g. Finally, the samples were left to air dry naturally at room temperature and atmospheric pressure, allowing the sulfuric acid to react thoroughly with the samples to obtain the modified nonwovens.

In situ growth of MOF-303 on the pre-treated nonwovens. Firstly, 720 mL of deionized water (solvent), 10.4 g of AlCl_3_, and 7.5 g (43.08 mmol) of H_3_PDC·H_2_O were transferred into a 1 L glass container and stirred under the condition of a water bath until completely dissolved, which was recorded as solution A. A total of 2.6 g (65 mmol) of NaOH was dissolved in 30 mL of deionized water, denoted as solution B. Then, the modified nonwovens were put into solution A, and solution B was added drop by drop to solution A. The above mixture was sealed and reacted at 100 °C for 24 h. Finally, the obtained microfiber nonwovens with a gradient asymmetric structure for a microfiber synthetic leather base were washed with deionized water and methanol three times, ultrasonically for 2 h, and dried in a vacuum oven.

### 3.3. Characterization

The surface morphology of the microfiber nonwovens was evaluated via scanning electron microscope (SEM; S-4800, Hitachi, Tokyo, Japan) coupled with an energy-dispersive spectroscopy (EDS) unit operated at an acceleration voltage of 15 kV. In order to enhance electric conductivity, the samples were sputtered with gold ahead of SEM measurement. The fiber diameter and distribution of the fabricated microfiber nonwovens were measured by nano measurer analysis software. 

A water contact angle test was performed using a contact angle meter (OCA20; Dataphysics, Filderstadt, Germany) equipped with a digital camera at room temperature, using 3 μL of water as an indicator. The test droplet was 3 μL. The water contact angle was tested when the droplet dropped on the surface for 1 s, and the droplet’s complete absorption time for the contact angle of 0° (droplet disappearance time) was recorded. At least five measurements were averaged to obtain a reliable value. 

The liquid strikethrough time of the microfiber nonwovens was tested using a liquid strikethrough test (YG(B) 231D; Darong, Tianjin, China). A drop of test solution was allowed to fall on the sample and the time taken for the solution to transport from the upper layer of the microfiber nonwovens to the inner layer was noted [27].

The water vapor absorption and desorption of microfiber nonwovens were measured by the gravimetric method in a Vacuum Vapor Sorption Analyzer (3H-2000PW; Beishide, Beijing, China) under 20 °C. For absorption, the relative humidity was increased and equilibrated stepwise from 5% to 95% at a 5% RH interval. For desorption, the atmospheric moisture content was decreased and equilibrated stepwise at 95% by up to 5%. The sample remained in each step until the moisture content change was less than 0.1 g for 1 h. The set of absorption–desorption isotherm test conditions is listed in Table 2.

The air permeability of the microfiber nonwovens was measured according to GB/T5453-1997 using an automatic air permeability tester (YG461H; Ningbo Textiles Instrument Co., Ltd., Ningbo, China) with a surface area of 20 cm^2^ and pressure drop of 100 Pa [28]. Ten specimens were measured per sample and the average value was reported.

The tensile strength of the microfiber nonwovens was determined using a tensile tester (Instron 3369; America Instron Co., Ltd., Norwood, MA, USA) in accordance with the GB/T 24218.3-2010 standard, with a cross-head speed of 100 mm/min [29]. The specimen width was 50 mm ± 0.5 mm, and the specimen length was 250 mm ± 0.5 mm. The clamping distance was 200 mm. The same group of specimens were tested five times to obtain the average value.

## 4. Conclusions

In summary, an asymmetrically wettable PET/PA6 HSMN was designed and constructed for a microfiber synthetic leather base. Due to its novel gradient wetting structure, the modified PET/PA6 HSMN was verified as possessing an improved hydrophilic property, liquid strikethrough performance, and adsorption capacity. Additionally, the modified PET/PA6 HSMN is qualified with high air permeability and tensile strength. This work provides new inspiration for creating PET/PA6 HSMNs with high hygiene performance and asymmetric wettability, with the prospect of preparing microfiber synthetic leather for clothing, which is expected to advance synthetic leather.

## Figures and Tables

**Table 1:** EDS data of the microfiber nonwovens treated with sulfuric acid at different concentrations and MOF-303.

Element	Atomic Percent/%				
	0%+ MOF-303	5%+ MOF-303	10%+ MOF-303	15%+ MOF-303	20%+ MOF-303
C	74.66	68.14	56.95	57.85	65.07
O	22.62	30.57	33.81	34.19	27.33
N	2.14	0.72	7.71	5.33	3.83
Al	0.58	0.98	1.74	2.76	4.56

**Table 2:** Set of absorption–desorption isotherm test conditions.

Sample	Microfiber Nonwovens: (a) Untreated PET/PA6 HSMN (b) PET/PA6 HSMN Treated with Sulfuric Acid of 20% and MOF-303				
Test temperature	20 °C	Saturated vapor pressure	2.337 kPa	Adsorbent	Water vapor
Degassing method	Heating + constant pressure	Pre-treatment temperature	150 °C	Pre-treatment time	180 min
Carrier gas	Nitrogen gas	Adsorption equilibrium conditions	0.1 mg/h	Total flow rate	400 sccm
Pressure point	5%, 10%, 15%, 20%, 30%, 50%, 70%, 95%				

## Data Availability

The data that support the findings of this study are available from the author (B.Z.) upon reasonable request.
